# Biopsy of the Superficial Sensory Radial Nerve in the Distal Forearm: A Technical Note

**DOI:** 10.7759/cureus.98796

**Published:** 2025-12-09

**Authors:** Alexander E Shearin, Daniel Surdell

**Affiliations:** 1 Neurological Surgery, University of Nebraska Medical Center, Omaha, USA

**Keywords:** distal forearm, nerve biopsy, nerve surgery, superficial radial nerve, upper extremity neuropathy

## Abstract

Neurosurgeons are frequently consulted for nerve biopsy. Common indications for nerve biopsy include vasculitis, peripheral neuropathy of unknown etiology, amyloidosis, and chronic inflammatory demyelinating polyneuropathy (CIDP). While the sural nerve is most commonly selected, a specimen from the superficial sensory radial nerve (SSRN) is often chosen when the pathology is confined to the upper extremities. The SSRN is typically exposed in the proximal forearm between the brachioradialis (BR) and extensor carpi radialis longus (ECRL) tendons. Here, we describe the technical details for biopsy of the SSRN in the distal forearm, where the nerve is superficially located.

## Introduction

Neurosurgeons are often consulted to perform a nerve biopsy in cases of peripheral neuropathy. Suspected pathologies for which nerve biopsy may be necessary for diagnosis include vasculitis, amyloidosis, peripheral neuropathy of unknown etiology, and chronic inflammatory demyelinating polyneuropathy (CIDP). The sural nerve is typically chosen for biopsy, given its pure sensory function, superficial location, and relatively small reported area of diminished sensation following biopsy [1]. When a nerve biopsy is requested at our institution with no specifications, it is our preference to biopsy the sural nerve due to the small expected area of sensory loss or paresthesias. When the pathology in question disproportionately affects the upper extremities, the superficial sensory radial nerve (SSRN) may be selected instead [2]. The SSRN is a pure sensory nerve originating from the bifurcation of the radial nerve into the posterior interosseous nerve (PIN) and SSRN; it courses over the supinator muscle and between the brachioradialis (BR) and extensor carpi radialis longus (ECRL) muscles, piercing the antebrachial fascia in the distal third of the forearm to become subcutaneous and supply a variable sensory area over the radial half of the dorsum of the hand [3]. In reviewing articles and textbook chapters that describe SSRN exposure, most focused on accessing the nerve in the proximal forearm between the BR and ECRL [4-8] or as it emerges from the superficial fascia adjacent to the BR tendon [2], we found some sources that describe exposure of the nerve in the distal forearm and wrist [9,10] and some with a textual description of SSRN exposure in that distal location with no figures [6,11]. While these latter sources describe access to the SSRN in the location of interest, they omit the technical details to fully describe the procedure. Our purpose here is to review these details for the biopsy of the SSRN in the distal forearm.

## Technical report

**Figure 1:**
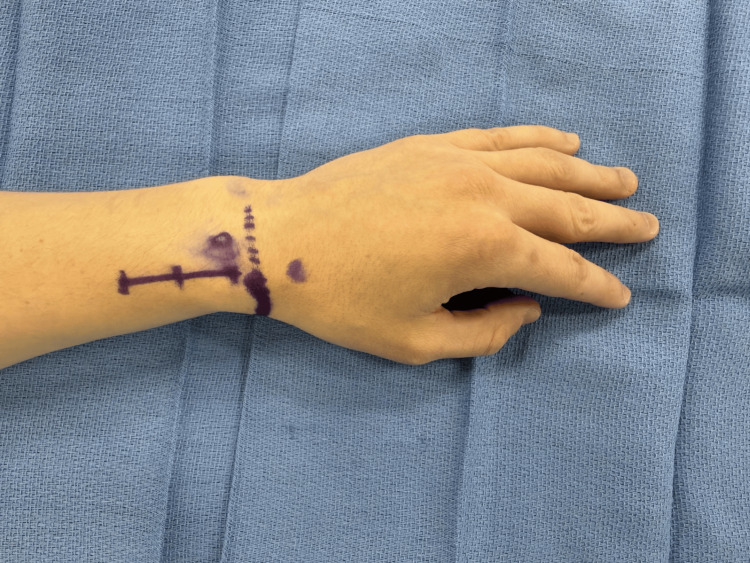
Landmarks and marking the incision In the preop area, the dorsal tubercle of the radius (circle), radial styloid process (thick, solid line), and extensor crease of the wrist (dotted line) are marked. A 3.5-4 cm incision parallel to the radius between the dorsal tubercle and styloid is planned.

**Figure 2:**
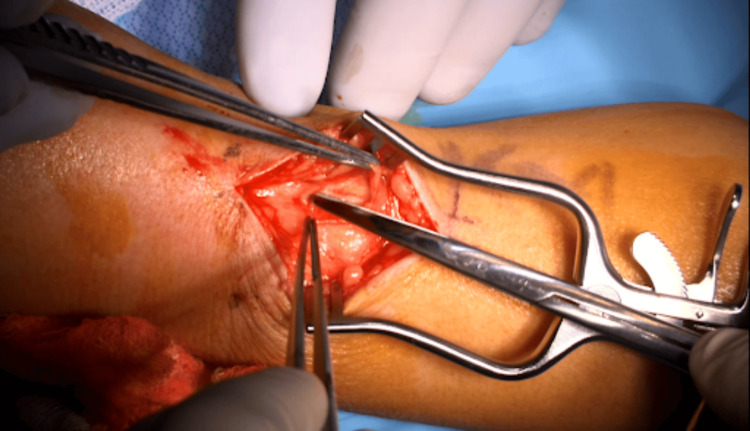
Subcutaneous dissection After the skin incision, dissecting scissors are used to further divide the subcutaneous tissues. For orientation, this is the patient's left arm with the hand off to the left of the image and the more proximal forearm to the right. The thumb can be seen in the upper left part of the image.

**Figure 3:**
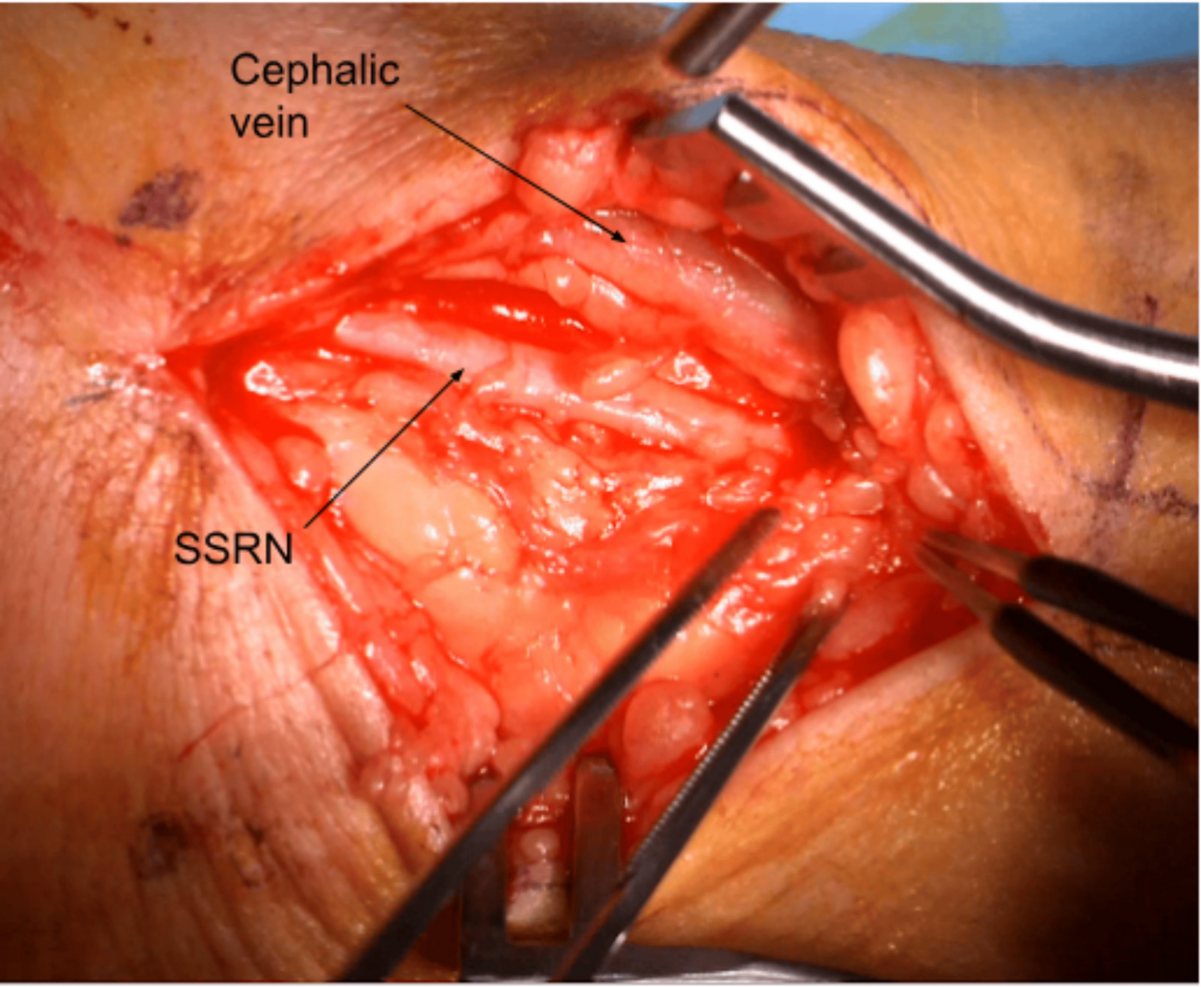
Identification of the superficial sensory radial nerve The SSRN is identified within the subcutaneous tissue dorsal (medial) to the cephalic vein. SSRN: Superficial sensory radial nerve

**Figure 4:**
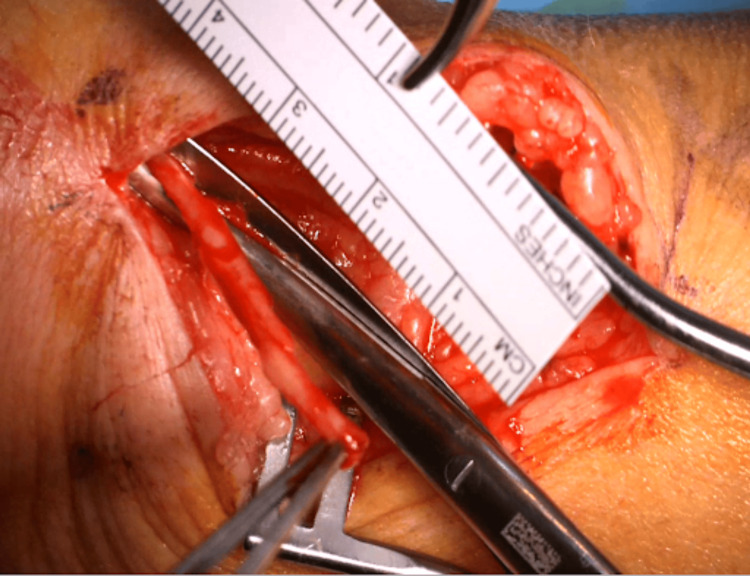
Nerve biopsy Sufficient length of nerve has been measured for the biopsy (3 cm). The nerve is cut first proximally and then distally to complete the biopsy.

**Figure 5:**
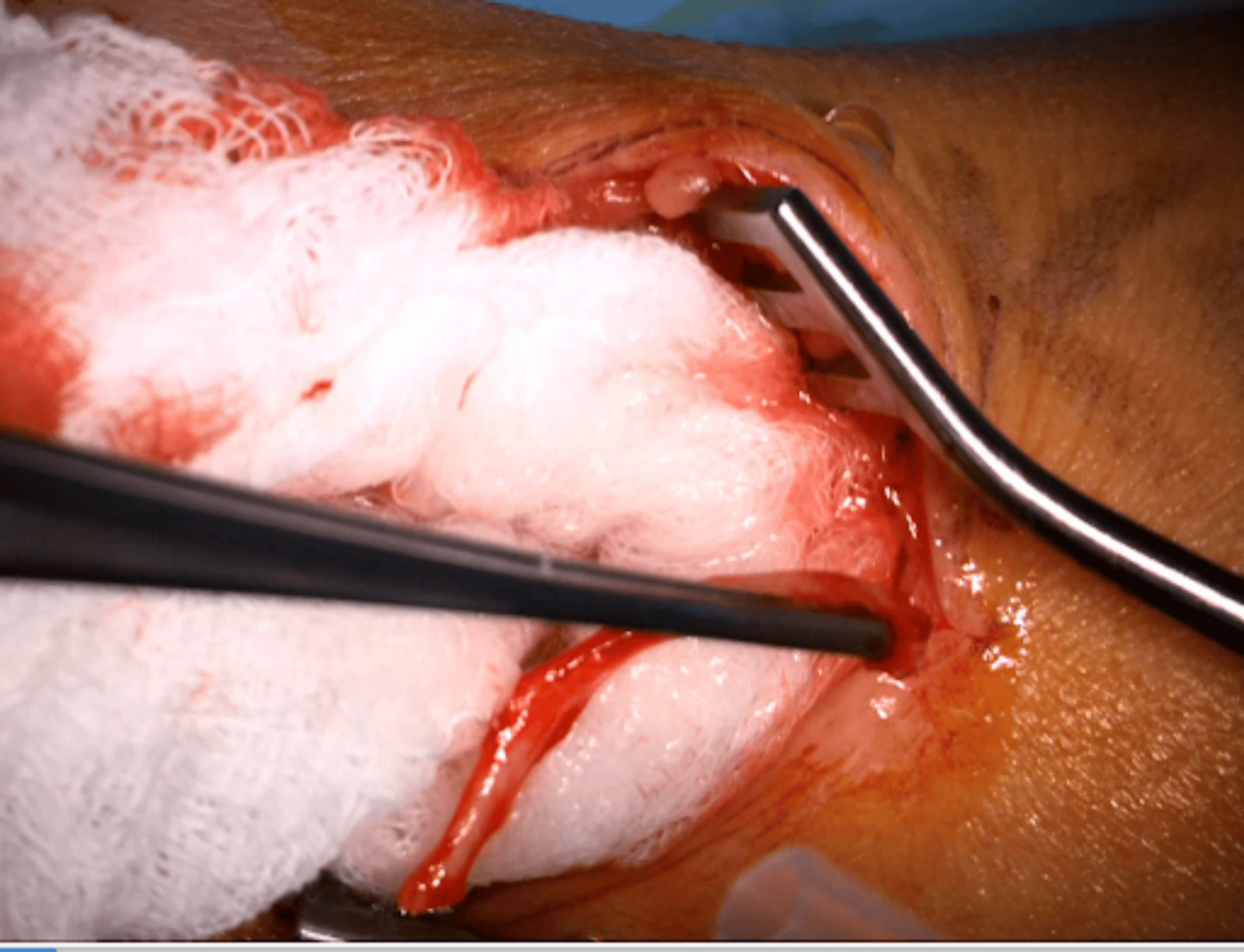
Specimen The SSRN biopsy is completed, and the specimen is sent to pathology. The field is flooded, and a lap sponge is placed into the wound. SSRN: Superficial sensory radial nerve

For our procedure, the patient is seen in the preoperative area where the following landmarks are identified and marked: (1) radial styloid process, (2) dorsal tubercle of the radius, (3) extensor wrist crease (Figure 1). These landmarks are chosen based on a cadaveric study, which was intended to define a safe area of incision for de Quervain tenosynovitis by delineating the relationships between the distal SSRN, cephalic vein, radial artery, and first compartment of the extensor retinaculum [12]. In the operating room, the patient is positioned supine with the arm abducted, resting on an arm board halfway between pronation and supination. IV antibiotics are given prior to starting the procedure. The planned incision spans approximately 3.5-4 cm parallel to the radius and is in the middle of the interval between the dorsal tubercle and radial styloid. Should this incision clearly put the cephalic vein at risk, it can be moved slightly dorsal or ventral. Local anesthetic (lidocaine with epinephrine 1:100,000) is infiltrated superficially along the planned incision. An Escmarch bandage is used to exsanguinate the arm, and a tourniquet is inflated to 250 mm Hg. The Esmarch is removed once the tourniquet is inflated. The incision is then made to the level of the subcutaneous tissue, followed by placement of a self-retaining retractor. Sharp dissection through the subcutaneous tissue proceeds with tenotomy scissors (Figure 2). The SSRN is found within the subcutaneous tissue in close proximity to the cephalic vein, to which the nerve is dorsal in this case (Figure 3). After identifying the nerve, it is circumferentially dissected free from the surrounding tissues, and any smaller, tethering branches coming off the nerve are divided. 3 cm of nerve is measured and cut proximally, then distally (Figure 4). After the specimen is removed from the field and sent for pathology, the wound is copiously irrigated (Figure 5). A lap sponge is used to hold pressure on the wound for 5 minutes after the tourniquet is deflated. Final hemostasis is then achieved with bipolar electrocautery. The skin is closed in a single layer with multiple interrupted nylon sutures, and a lightly compressive dressing is applied and left in place for 2 days. The sutures are typically removed two to three weeks following the procedure in the clinic.

## Discussion

Biopsy of the SSRN is an option in cases of peripheral neuropathy primarily affecting the upper extremities when tissue is required for diagnosis. While methods describing the SSRN biopsy have typically focused on accessing the nerve in the proximal forearm between the BR and ECRL, we describe the technical details for accessing the nerve in the distal forearm. The theoretical benefits to this approach, as compared to accessing the nerve more proximally in the forearm, include a more superficial location of the nerve requiring less dissection and thus potentially shorter operating times. Another reason we choose to biopsy the nerve distally in the forearm is based on the observation by Robson et al that the SSRN divides into multiple branches as it nears the hand, with the first branch coming off the SSRN trunk on average 5 cm proximal to the radial styloid [12]. Biopsy of the nerve distal to this branching thus has the potential to spare some of the SSRN sensory territory, whereas a more proximal biopsy might not. The only study to our knowledge comparing SSRN territory sensation before and after biopsy is from 1971, in which patients with leprosy had fascicular biopsy through a transverse incision at the wrist, which showed long-term worsening of sensation in the SSRN territory in only 2 of 59 patients [13]. This study is not broadly applicable to our case due to the differences in suspected pathology-we have never received a request for nerve biopsy in a patient with leprosy-as well as the technique in that we perform whole nerve rather than fascicular biopsy. Without data to support biopsy at one location over the other in terms of sensory preservation, we prefer the distal location to at least attempt to preserve a branch of the SSRN in continuity.

Potential complications related to the anatomy in this location include vascular injury to the radial artery or cephalic vein, and accidental biopsy or neural injury to the lateral antebrachial cutaneous nerve (LABCN). According to the cadaveric study of Robson et al, the cephalic vein is closely associated with (that is, within 2 mm of) the SSRN in 80% of specimens; the radial artery was closely associated with the nerve in 48% [12]. It should be noted that the proximity of the radial artery to the nerve, while present in nearly half of cases, appears, based on their photographs, to primarily apply to the first branch of the SSRN rather than the main trunk that continues distally [12]. It is partly in the hope of preserving this branch that our procedure is based in the distal forearm; hence, we generally think of the radial artery as being less at risk. Thus, accidental injury to the cephalic vein is the most likely vascular complication of SSRN biopsy in the distal forearm. Injury to the LABCN may occur as there is anatomic and sensory territory overlap frequently present between it and the SSRN [14,15]. The usual possible complications related to nerve biopsy include the development of neuropathic pain or a painful neuroma, postoperative hematoma, seroma, wound infection, and obtaining an inadequate specimen.

Based on the study by Hilton et al, which combined a single surgeon case series with cumulative data from other series reporting on complications of sural and peroneal nerve biopsies, the most common complications were paresthesia (40%), dysesthesia (33%), postoperative pain (30%), and wound infection (8%) [1]. For the SSRN, the distribution of sensory loss, paresthesias, or pain would involve a variable area comprising the radial half of the dorsum of the hand, including the thumb and first and second digits proximal to the distal phalanx, as well as a small area of the lateral aspect of the thenar eminence [16]. While there are no similarly large studies documenting complications arising from sensory radial nerve biopsy, we use this data in our practice to counsel patients regarding the risks of complications of nerve biopsy more generally. Given that the expected diagnostic yield for nerve biopsy in general is relatively low-63% in one series of 107 nerve biopsies [17]-it behooves the surgeon to ensure all other diagnostic avenues, such as laboratory and electrodiagnostic testing, have been exhausted prior to proceeding with nerve biopsy, considering the risk of complications.

## Conclusions

Nerve biopsy is often requested for diagnostic confirmation in cases of vasculitis, amyloidosis, peripheral neuropathy of unknown etiology, and CIDP. Biopsy of the SSRN is an alternative to the sural nerve when the pathology in question disproportionately affects the upper extremities. Accessing the SSRN distally in the forearm, where it is superficial, has the theoretical advantages of less dissection required compared to more proximal exposure, as well as the potential for sparing a small part of the SSRN sensory distribution by transecting the nerve distal to its first branch. The relevant neurovascular structures at risk of injury in this area include the LABC, cephalic vein, and radial artery. While no large series of complications of SSRN biopsy has been published, it is reasonable to counsel patients on the most common complications of nerve biopsy, which include postoperative pain, paresthesia, dysesthesia, and wound infection.
